# Newly developed magnifying endoscopic classification of the Japan Esophageal Society to identify superficial Barrett’s esophagus-related neoplasms

**DOI:** 10.1007/s10388-018-0623-y

**Published:** 2018-06-19

**Authors:** Kenichi Goda, Junko Fujisaki, Ryu Ishihara, Manabu Takeuchi, Akiko Takahashi, Yasuhiro Takaki, Dai Hirasawa, Kumiko Momma, Yuji Amano, Kazuyoshi Yagi, Hiroto Furuhashi, Tomoki Shimizu, Takashi Kanesaka, Satoru Hashimoto, Yoichiro Ono, Taku Yamagata, Junko Fujiwara, Takane Azumi, Masako Nishikawa, Gen Watanabe, Yasuo Ohkura, Tsuneo Oyama

**Affiliations:** 1 Digestive Diseases Center, Showa University Koto Toyosu Hospital, 5-1-38 Toyosu, Koto-ku, Tokyo, 135-8577 Japan; 2 Department of Gastroenterology, Japanese Foundation for Cancer Research, Cancer Institute Hospital, Tokyo, Japan; 3 Department of Gastrointestinal Oncology, Osaka International Cancer Institute, Osaka, Japan; 4 Department of Gastroenterology, Nagaoka Red Cross Hospital, Niigata, Japan; 5 Department of Endoscopy, Saku Central Hospital Advanced Care Center, Nagano, Japan; 6 Department of Gastroenterology, Ashiya Central Hospital, Fukuoka, Japan; 7 Department of Gastroenterology, Sendai Kousei Hospital, Miyagi, Japan; 8 Department of Endoscopy, Tokyo Metropolitan Cancer and Infectious Disease Center Komagome Hospital, Tokyo, Japan; 9 Department of Endoscopy, New Tokyo Hospital, Chiba, Japan; 10 Department of Gastroenterology and Hepatology, Uonuma Institute of Community Medicine, Niigata University Medical and Dental Hospital, Niigata, Japan; 11 Department of Endoscopy, The Jikei University School of Medicine, Tokyo, Japan; 12 Division of Gastroenterology and Hepatology, Graduate School of Medical and Dental Sciences, Niigata University, Niigata, Japan; 13 Department of Gastroenterology, Fukuoka University Chikushi Hospital, Fukuoka, Japan; 14 Department of Gastroenterology, Sendai City Medical Center, Miyagi, Japan; 15 Department of Gastroenterology, International University of Health and Welfare Ichikawa Hospital, Chiba, Japan; 16 Clinical Research Support Center, The Jikei University School of Medicine, Tokyo, Japan; 17 Department of Pathology, Niigata Cancer Center Hospital, Niigata, Japan; 18 Pathology and Cytology Center, PCL Japan, Saitama, Japan

**Keywords:** Barrett’s esophagus, Esophageal adenocarcinoma, Endoscopic classification, Magnification endoscopy, Narrow band imaging, Japan Esophageal Society

## Abstract

**Aim and methods:**

The Japan Esophageal Society created a working committee group consisting of 11 expert endoscopists and 2 pathologists with expertise in Barrett’s esophagus (BE) and esophageal adenocarcinoma. The group developed a consensus-based classification for the diagnosis of superficial BE-related neoplasms using magnifying endoscopy.

**Results:**

The classification has three characteristics: simplified, an easily understood classification by incorporating the diagnostic criteria for the early gastric cancer, including the white zone and demarcation line, and the presence of a modified flat pattern corresponding to non-dysplastic histology by adding novel diagnostic criteria. Magnifying endoscopic findings are composed of mucosal and vascular patterns, and are initially classified as “visible” or “invisible.” Morphologic features were evaluated for “visible” patterns, and were subsequently rated as “regular” or “irregular,” and the histology, non-dysplastic or dysplastic, was predicted.

**Conclusion:**

We introduce the process and outline of the magnifying endoscopic classification.

## Introduction

Barrett’s esophagus (BE)-related neoplasms, including esophageal adenocarcinoma (EAC), are still rare in Japan [1, 2]. However, the increasing number of patients with EAC because of the increasing prevalence of gastroesophageal reflux disease and BE is concerning [3]. Early detection is crucial for good quality of life and favorable prognosis of patients with EAC. Superficial BE-related neoplasms (SBN), including dysplasia and particularly EAC with flat macroscopic type, are often difficult to detect by white light endoscopy alone [4, 5]. Studies showed the utility of magnifying endoscopy to detect SBN and proposed several magnifying endoscopic classifications based on narrow-band imaging (NBI) findings [6–9]. However, these classifications involve complicated and diverse criteria, making them difficult to use in clinical practice by general endoscopists.

We, the committee members of the Japan Esophageal Society (JES), developed a new magnifying endoscopic classification of the JES for predicting the histology of Barrett’s epithelium, and named it as JES-BE classification. We introduce the process and outline of the JES-BE classification system.

## Working group

The JES created a working committee group in 2012 consisting of 11 expert endoscopists and 2 pathologists with expertise in gastrointestinal neoplasms, including SBN, who are from 10 domestic institutions of a high-volume center or an academic university hospital. The working committee members were assembled during the 66th annual meeting of the JES and convened nine times to proceed with the creation, assessment, and examination of the JES-BE classification system.

## Development of consensus-based classification system

The JES working group collected high-definition magnification NBI (HM-NBI) images of 20 cases of non-dysplastic BE, including specialized intestinal metaplasia (SIM), and 25 cases of dysplastic BE (i.e., SBN, including low- and high-grade dysplasia, and flat-type superficial EAC invading up to the submucosa) from 10 domestic hospitals of the working group members. The working group discussed about mucosal and vascular patterns of the images and histologic findings at the mucosal site where the images had been obtained. The working group developed the JES-BE classification system based upon consensus among the working committee members.

## The JES-BE classification system

Table 1 shows the JES-BE classification system. The mucosal and vascular patterns in each HM-NBI image were initially classified as “visible” or “invisible”. The “invisible” pattern cannot be subclassified. The “visible” mucosal and vascular patterns were subclassified as “pit” or “non-pit” and “net” or “non-net”, respectively. The “pit” type mucosal pattern was marked by a circular pattern, and the “non-pit” was marked by tubular, linear, or ridged/villous patterns (Table 3). The “net” type vascular pattern was marked by a network of vessel formation connected to each other, and the “non-net” type was marked by vessels without network formation [10]. Finally, each pattern was classified as either “regular” or “irregular” based on the diagnostic criteria agreed upon by the working group. The diagnostic criteria for regularity are listed in Table 2.

**Table 1:** New magnifying endoscopic classification of the Japan Esophageal Society for predicting histology of Barrett’s epithelium (JES-BE classification)

	Visibility	Morphologic features		Regularity^a^	Predicted histology
Mucosal pattern	Visible	Pit	Circular or round	Regular or Irregular	Non-dysplastic or Dysplastic
		Non-pit	Ridged, villous, linear, or tubular		
				Unclassified	Dysplastic
	Invisible^b^	–	–	–	–
Vascular pattern	Visible	Net	Network formation	Regular or Irregular	Non-dysplastic or Dysplastic
		Non-net^c^	Not forming network		
				Unclassified	Dysplastic
	Invisible	–	–	–	–

^a^Classified based on the diagnostic criteria for regularity (listed in Table 2)

^b^Including a flat pattern

^c^Including normal-appearing long-branching vessels and greenish thick vessels suggestive of a flat pattern

**Table 2:** Diagnostic criteria for the irregularity of mucosal and vascular patterns, including the modified flat pattern

	Diagnostic criteria for regularity		Predicted histology
	Mucosal pattern	Vascular pattern	
Regular pattern	Form/size: similar	Form: similar or bending and branching gently or regularly	Non-dysplastic
	Arrangement: regular	Caliber change: gradual	
	Density: low same as surrounding area	Location: between or in mucosal patterns	
	White zone: clearly visible and/or with homogeneous width		
Flat pattern^a^	Completely flat surface (i.e. invisible mucosal pattern) without a clear demarcation line	Greenish thick vessels and/or long branching vessels	
Irregular pattern^b^	Form/size: various	Form: various or bending and branching steeply or irregularly	Dysplastic (LGD/HGD/superficial EAC)
	Arrangement: irregular	Caliber change: abrupt	
	Density: high	Location: beyond of regardless of mucosal patterns	
	White zone: obscure/invisible or heterogeneous width		

*LGD* low-grade dysplasia, *HGD* high-grade dysplasia, *superficial EAC* esophageal adenocarcinoma in which to invade up to the submucosa

^a^Modified criteria for a flat pattern

^b^Irregular pattern included unclassified pattern

Regularity of mucosal pattern is evaluated based on form, size, arrangement, density, and white zone. The white zone has been used in the M-NBI diagnosis of the early gastric cancer. Yagi et al. [11] initially mentioned a white zone that can be seen as a whitish edge of mucosal pattern of the early gastric cancer under magnification NBI observation. They described the hypothesis of a mechanism of white zone visibility. They suggested that cancerous lesions often showed indistinct or invisible white zone that would correspond to absent surface pattern and disappearance of the fine mucosal structure in the previous studies on the early gastric cancer [12, 14]. We, the working group members, reached a consensus that clearly visible and indistict or invisible white zones would be suggestive of non-cancerous and cancerous lesions, respectively.

Regular mucosal patterns were marked by showing similar forms and/or homogeneous sizes, regular arrangement, and clearly visible white zone. Irregular mucosal patterns were marked by various forms, heterogeneous sizes, irregular arrangement, high density, and indistinct or invisible white zone [11–13].

Regular vascular patterns were marked by their location between or along mucosal ridges with similar forms, gently bending, regularly branching, and gradual changes in the vessel’s caliber. Irregular vascular patterns were marked by not following mucosal structures with various forms, steeply bending, irregularly branching, and abrupt changes in the vessel’s caliber [6, 12, 13].

If magnifying endoscopic findings would show both of regular and irregular patterns, the irregular pattern was representative of the endoscopic findings to prevent form missing the chance of biopsy for a suspected dysplastic lesion.

“Unclassified” mucosal or vascular pattern that could not be classified into “regular” or “irregular” was rated as an “irregular” pattern because tissue samples from mucosal sites with “unclassified” pattern should be obtained for biopsy, similar to that with “irregular” pattern (Table 2).

Flat pattern was originally defined as flat mucosa (none of pits and villi: i.e., absent pattern) with normal-appearing long-branching vessels in a previous study [6]. The original flat pattern was classified into a “regular” pattern and suggested to be of non-dysplastic BE, including SIM. The flat mucosal pattern and the long-branching vessels will mimic an absent mucosal pattern and often coexisted with steeply bending or tortuous vessels appearing an irregular vascular pattern, respectively. Both latter M-NBI patterns were suggestive of the early gastric cancer [12, 14]. Thus, interpretation of the flat pattern as a regular pattern that corresponds to non-dysplastic (i.e., non-cancerous) histology will be difficult, particularly among endoscopists with expertise in the M-NBI diagnosis of the early gastric cancer. A previous study showed that a distinct demarcation line was a primarily important diagnostic criterion for the early gastric cancer [14]. Our study demonstrated that none of non-dysplastic lesions with flat pattern have a distinct demarcation line [15]. In addition, greenish thick vessels are frequently seen in non-dysplastic lesion with flat pattern. Following these results, “no clear demarcation” and “a greenish thick vessel” were incorporated in the definitions of a modified flat pattern in this classification. We classified the modified flat pattern independently as “regular” mucosal/vascular patterns (Table 2).

## Predicted histology based on JES-BE classification

**Fig. 1:**
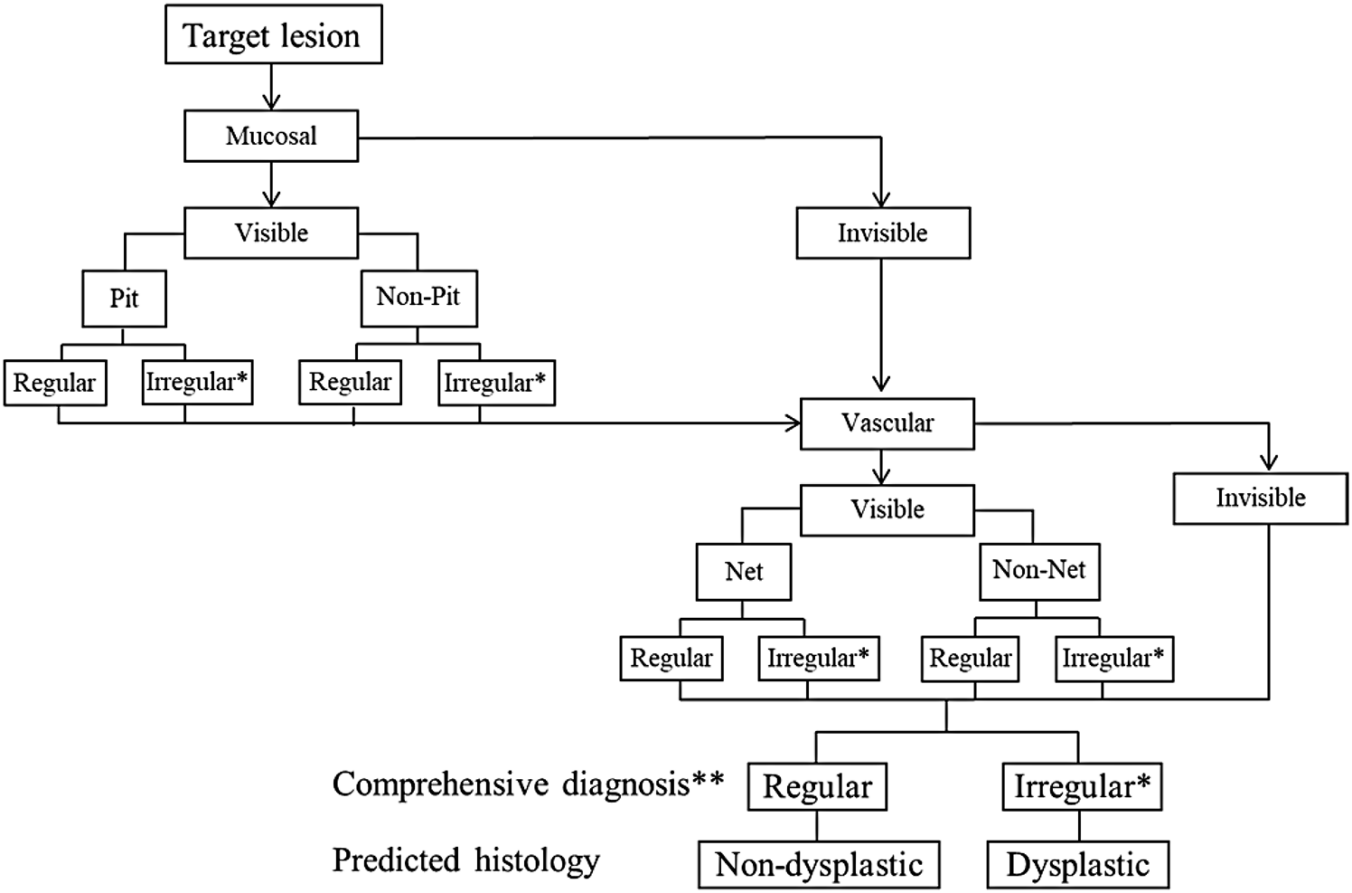
Diagnostic flowchart using the JES-BE classification. *Irregular pattern including unclassified pattern. **When the mucosal/vascular patterns are visible and graded differently (i.e., one regular and other irregular or invisible), predicted histology is determined based on a comprehensive diagnosis

Visible mucosal/vascular patterns allow histology prediction, whereas invisible mucosal/vascular patterns do not. Regular and irregular mucosal/vascular patterns are suggestive of non-dysplastic and dysplastic histology, respectively. Unclassified mucosal/vascular patterns are suggestive of dysplastic histology. Flat pattern that meets modified criteria of this classification is rated as a regular pattern and suggestive of non-dysplastic histology. When the mucosal/vascular patterns are visible and graded differently (i.e., one regular and other irregular or invisible), predicted histology is determined non-dysplastic or dysplastic based on a comprehensive diagnosis of regular or irregular (Fig. 1).

Figure 1 shows a diagnostic flowchart based on this classification. If a lesion was found, we initially assess a mucosal pattern that would be visible under moderately to fully magnifying observation. We then assess a vascular pattern that would be visible under fully magnifying observation. Based on mucosal and vascular pattern, we finally make comprehensive diagnosis and predict histology.

## Relationships between JES-BE and other classifications (Table 3)

Recently, published NBI classification of BE classified absent mucosal pattern and normal-appearing long-branching vessels as “irregular” and “regular”, respectively [13]. Both patterns were the original criteria for a flat pattern [6]. To resolve the discrepancy, a flat pattern is independently classified as “regular” mucosal/vascular patterns by the modified diagnostic criteria in the JES-BE classification (Tables 2, 3). The JES-BE classification will be simpler than well-known traditional classifications **[**6, 7, 9].

**Table 3:** Relationships between currently known and newly developed classifications of surface patterns for predicting histology of Barrett’s epithelium

	Kara et al. [6]		Sharma et al. [7]	Anagnostopoulos et al. [9]	BING [13]	JES-BE	
Mucosal pattern	Flat		–	Absent (flat or non-structural)	Irregular	Flat^b^	Regular^c^
	Villous or gyrus		Circular	Round	Regular	Pit or non-pit	
			Ridge/villous	Linear/tubular/villous			
	Irregular		Irregular/distorted	Irregular	Irregular		Irregular^c^
Vascular pattern	Regular^a^	Absent of ABV	Normal	Regular	Regular	Flat^b^	Regular^c^
	Irregular	Present of ABV	Abnormal	Irregular	Irregular	Net or Non-net	Irregular^c^

*ABV* abnormal vessels, *BING* Barrett’s international narrow-band imaging group

^a^Including normal-appearing long-branching vessels that is an original diagnostic criterion for a flat pattern

^b^Flat pattern defined by modified criteria

^c^Regularity marked by diagnostic criteria (Table 2)

**Fig. 2:**
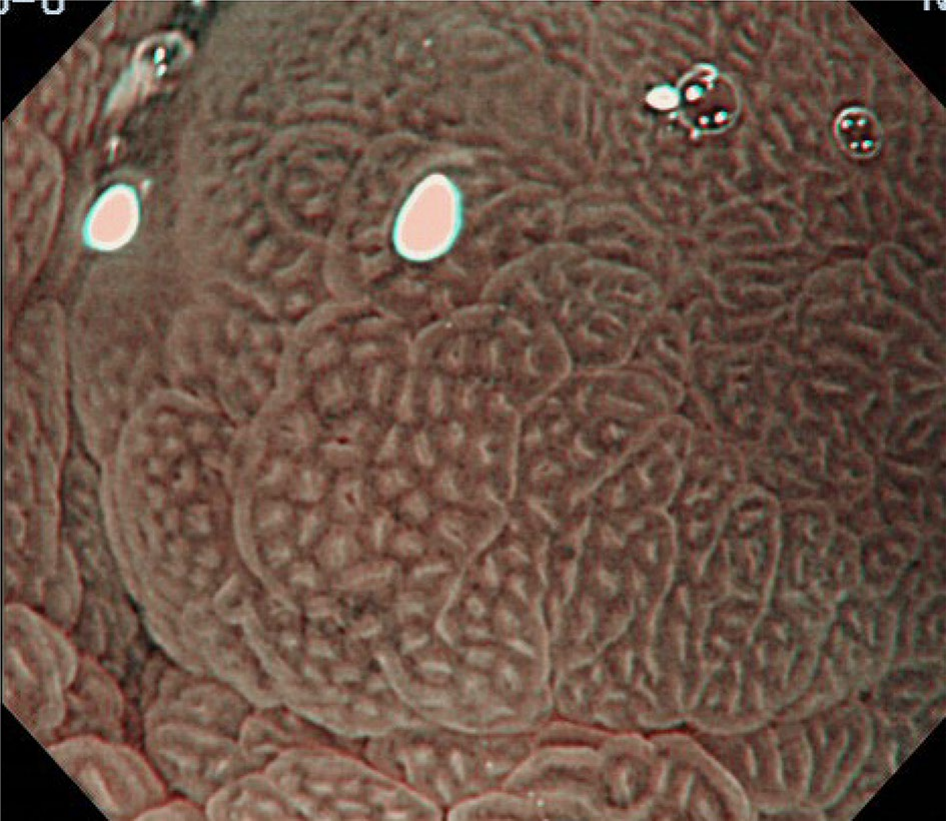
Magnifying NBI showing visible mucosal (pit type) and vascular patterns (net type) suggestive of regular patterns and predicted to be of non-dysplastic histology. Histology of the biopsy specimen obtained from the captured site demonstrated columnar epithelium including a parietal cells and chief cells (fundic type)

**Fig. 3:**
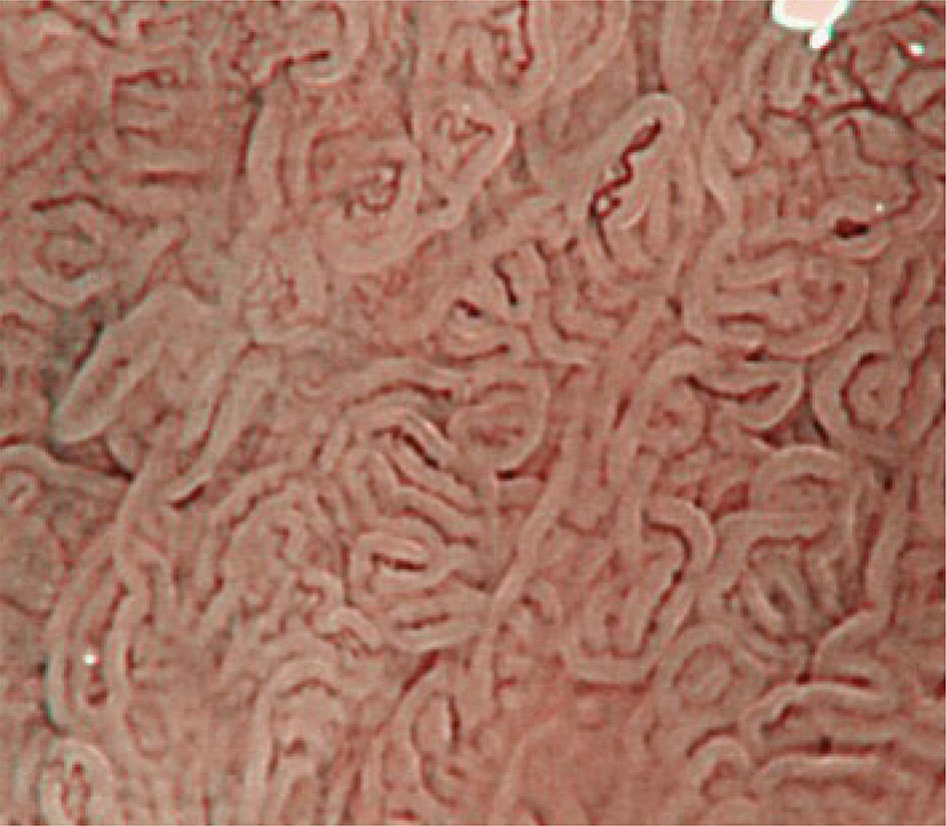
Magnifying NBI showing visible mucosal (non-pit type) and visible vascular patterns (non-net type) suggestive of regular patterns and predicted to be of non-dysplastic histology. Histology of the biopsy specimen obtained from the captured site demonstrated columnar epithelium including a cardiac type containing mucous-secreting columnar cells

**Fig. 4:**
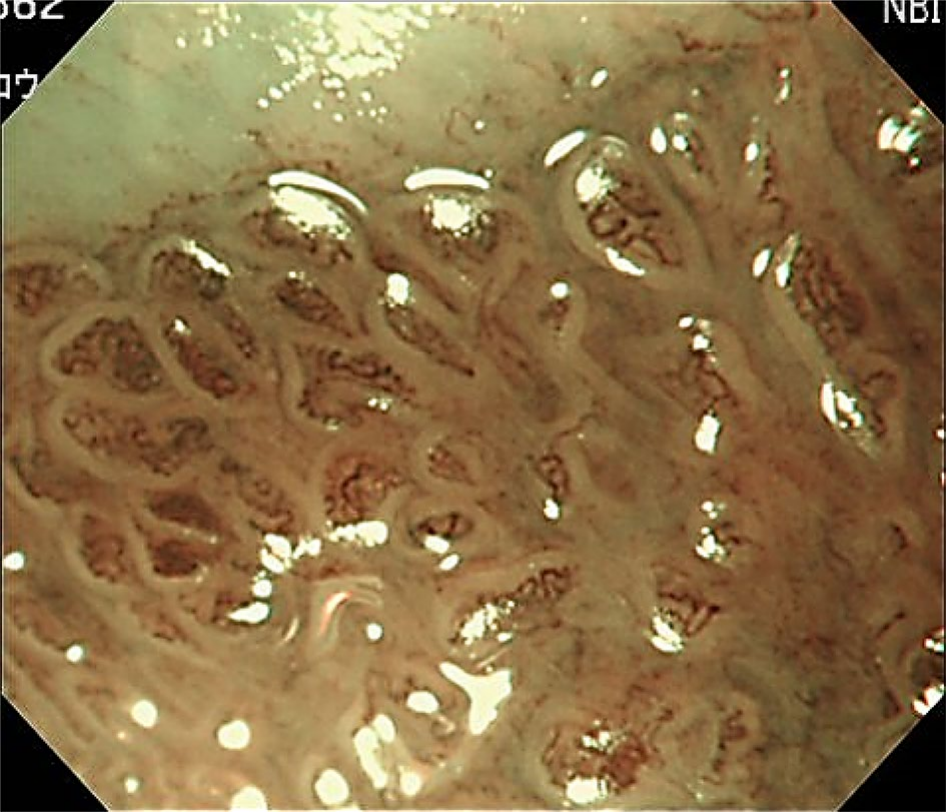
Magnifying NBI showing visible mucosal (non-pit) and visible vascular patterns (non-net type) are suggestive of irregular patterns, because the mucosal pattern shows villous patterns with various sizes and heterogenous width of white zones, and the vascular pattern bending and branching steeply or irregularly and locating beyond of regardless of mucosal patterns. A magnifying NBI is predicted to be of dysplastic histology and histology of the biopsy specimen obtained from the captured site demonstrated differentiated adenocarcinoma invading up to the superficial muscularis mucosa

**Fig. 5:**
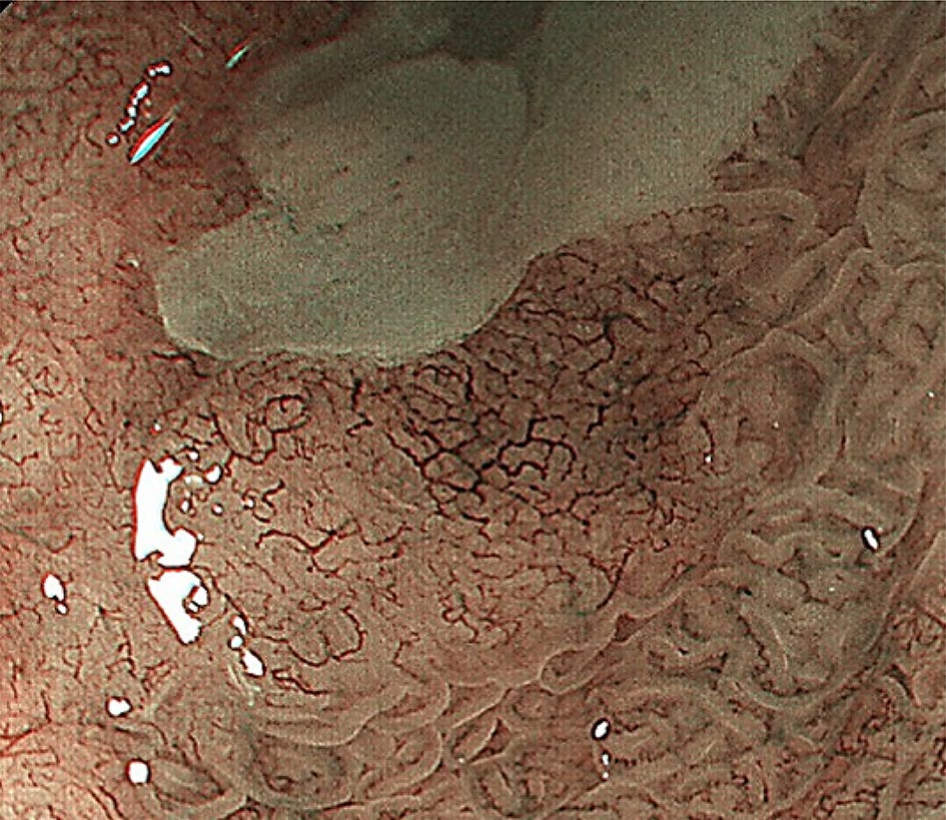
Magnifying NBI showing invisible mucosal and visible vascular patterns (net type) are suggestive of irregular patterns, because the vascular pattern demonstrates steep or irregular bending/branching vessels with abrupt caliber change. A magnifying NBI is predicted to be of dysplastic histology and histology of the biopsy specimen obtained from the captured site demonstrated differentiated adenocarcinoma invading up to the superficial muscularis mucosa

**Fig. 6:**
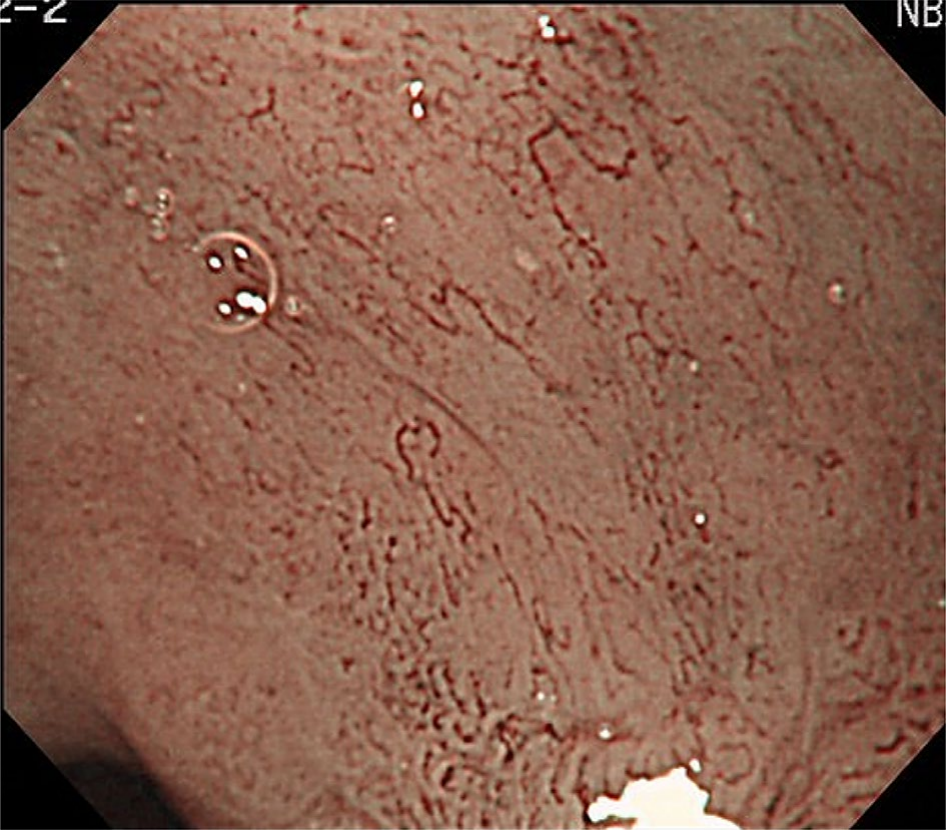
Magnifying NBI showing invisible mucosal and visible vascular patterns (non-net type) suggestive of an irregular pattern and predicted to be of dysplastic histology. Histology of the biopsy specimen obtained from the captured site demonstrated differentiated adenocarcinoma invading up to the deep muscularis mucosa. This captured area is completely flat and has no a greenish thick vessel

**Fig. 7:**
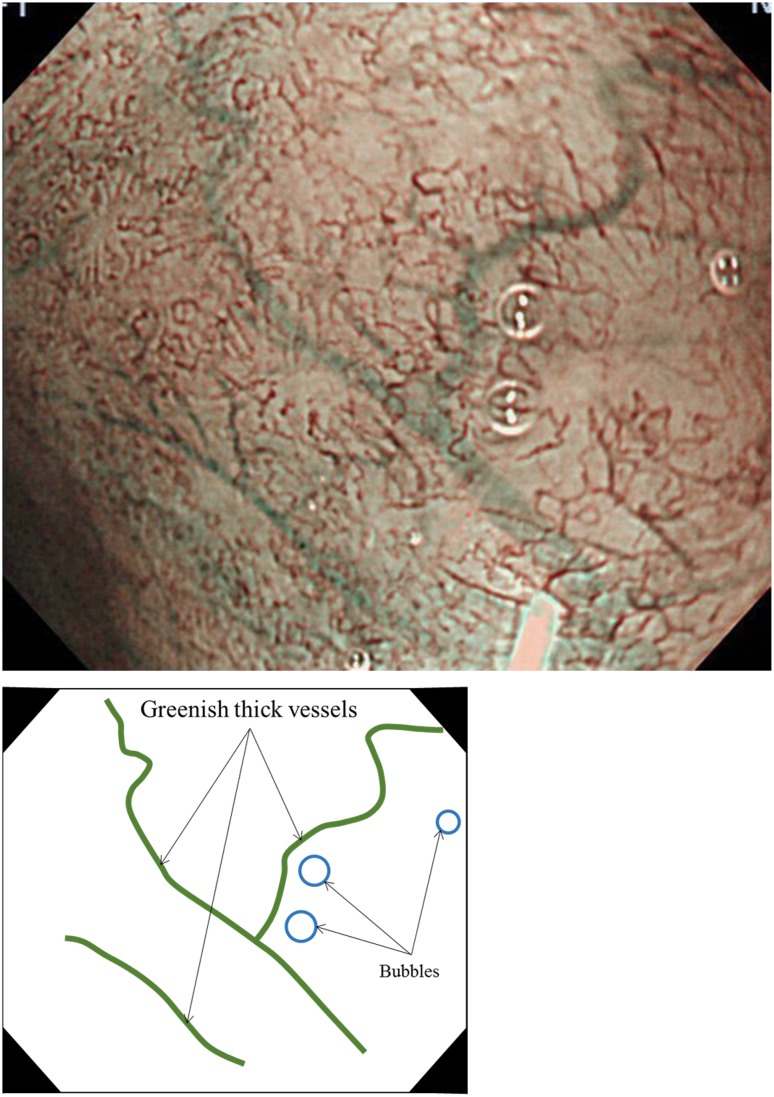
Magnifying NBI and the schema showing an invisible mucosal pattern as a completely flat surface and greenish thick vessels suggestive of a modified flat pattern and predicted to be of non-dysplastic histology. Histology of the biopsy specimen obtained from the captured site demonstrated columnar epithelium containing goblet cells corresponding to the specialized intestinal metaplasia

Figures 2, 3, 4, 5, 6, 7 show representative HM-NBI images with regular/irregular mucosal and vascular patterns.

## Conclusion

We developed the JES-BE classification and introduced the process and outline of the classification system. Further studies are needed to validate the diagnostic validity and reliability of the magnifying endoscopic classification.
